# A cross-sectional study on uric acid levels among Chinese adolescents

**DOI:** 10.1007/s00467-019-04357-w

**Published:** 2019-12-06

**Authors:** Jie Lu, Wenyan Sun, Lingling Cui, Xinde Li, Yuwei He, Zhen Liu, Hailong Li, Lin Han, Aichang Ji, Can Wang, Hui Zhang, Xiaopeng Ji, Wei Ren, Xuefeng Wang, Changgui Li

**Affiliations:** 1 Shandong Provincial Key Laboratory of Metabolic Diseases and Qingdao Key Laboratory of Gout, the Affiliated Hospital of Qingdao University, Qingdao, China; 2 Department of Endocrinology and Metabolism, the Affiliated Hospital of Qingdao University, Qingdao, China; 3 Institute of Metabolic Diseases, Qingdao University, Qingdao, 266003 China

**Keywords:** Prevalence, Hyperuricemia, Adolescents

## Abstract

**Background:**

The prevalence of hyperuricemia is increasing in adults, while the prevalence among adolescents is seldom reported.

**Methods:**

A cross-sectional survey by multistage, stratified sampling method was carried out in Shandong Province during 2017–2018. A total of 9371 adolescents aged from 13 to 19 years were randomly sampled and analyzed in this survey.

**Results:**

The overall mean serum uric acid (sUA) concentration was 6.08 ± 1.57 mg/dL and overall hyperuricemia prevalence was 25.4% and 60.5% (when hyperuricemia was defined as sUA ≥ 7 mg/dL or ≥ 5.5 mg/dL). Prevalence were 42.3% (male) and 8.0% (female) when limit was 7 mg/dL and prevalence were 82.1% (male) and 38.4% (female) when limit was 5.5 mg/dL. Male gender, increased body mass index, increased waist circumstance, increased triglycerides, increased fasting blood glucose, increased systolic blood pressure, decreased estimated glomerular filtration rate, and positive family gout history were associated with the enhanced risk of hyperuricemia according to univariate and/or multivariate logistic regression analysis. Food intake frequency of carbonate beverage, mutton, and other kinds varied between hyperuricemia adolescents and normal sUA ones.

**Conclusions:**

The studied adolescent population showed sUA level and hyperuricemia prevalence which are even higher than those of adults in China. The epidemic of youth hyperuricemia may pose a future threat of gout attacks and other hyperuricemia-related diseases, which alarms the public, health professionals and health policy makers to prepare the future health challenges.

## Introduction

Hyperuricemia is a major pathogenic factor for urate deposition diseases (gouty arthritis, renal disorder, etc*.*). In addition to gout, hyperuricemia is the risk factor of a series of diseases including ischemic heart disease, diabetes mellitus, lipid abnormalities, hypertension, stroke, and preeclampsia [1]. Gout and hyperuricemia affect predominantly males since serum uric acid (sUA) are considerably lower in women. Since gout is considered as an adult disease and hyperuricemia is asymptomatic in most people, pediatric hyperuricemia is often overlooked. Recent meta-analysis showed the prevalence of hyperuricemia and gout in mainland China reached 13.3% and 1.1% respectively [2]. However, it still lacks large sample-based reports on the prevalence of hyperuricemia among Chinese adolescents. The current study aimed to investigate the prevalence of hyperuricemia among adolescents (13–19 years old) from Shandong Province, one of the most populous provinces of China.

## Methods

In this study, we conducted a population-based cross-sectional study of enrolled students from the representative middle schools and universities of Shandong Province during 2017–2018. We used a multistage, stratified sampling method to obtain a representative sample of people aged 13–19 years in the general population. We randomly selected two urban and two rural districts (defined according to the National Bureau of Statistics in China) in Shandong Province. In the second stage, clusters of two schools were randomly selected from each district using probability proportional to size sampling. To minimize sampling bias due to age-dependent physiological change of urate level during puberty and life-style-related factors associated with the typical urban-rural dual system of China, we took stratified sampling strategy by matching the numbers of participants according to age and residence (rural or urban). In the third stage, individuals were randomly chosen (by simple randomization using SPSS software) from each school in each stratum. Only official registered students were enrolled and we excluded participants without blood samples and pregnant girls. Targeting participants were informed not to alter their diet or physical activity for at least 3 days before the examination. The study protocol was approved by the ethics review committee of the Affiliated Hospital of Qingdao University (#QYFY WZLL 25565). Participants 18 years or older provided written consent. Both written assent and parental permission were obtained in writing for youth younger than 18 years.

Body weight and height were measured according to a standard protocol. Body mass index (BMI) was calculated as weight in kilograms divided by height in meters squared. Blood samples were collected after overnight fasting. Serum levels of uric acid, creatinine, urea nitrogen, glucose, total cholesterol (TC), triglycerides (TG), and high-density lipoprotein-cholesterol (HDL-C) were measured enzymatically using an automatic analyzer (Toshiba, Tokyo, Japan) at the Gout Laboratory of the Affiliated Hospital of Qingdao University.

Since no previous standard of hyperuricemia for adolescents has been established, we defined hyperuricemia based on both putative adult hyperuricemia threshold (7.0 mg/dL) and hyperuricemia threshold among adolescents (5.5 mg/dL) reported previously [3]. To define the metabolic syndrome (MetS) in adolescents, we used a previously proposed modification of the definition by the International Diabetes Federation (IDF) [4]. Accordingly, obesity was defined as waist circumference ≥ 90th percentile among peer group, hyperlipidemia as triglycerides ≥ 1.7 mg/dL; hyperglycemia as fasting blood glucose ≥ 5.6 mmol/L. We defined hypertension as ≥ 130 mmHg systolic or ≥ 80 mmHg diastolic by guidelines of 2017 American Academy of Pediatrics (AAP) [5] for participants aged from 13 to 18 years and 2017 American College of Cardiology/American Heart Association (2017 ACC/AHA) [6] for 19-year-old adolescents. Estimated glomerular filtration rate (eGFR; ml/min per 1.73 m^2^) was calculated via the serum creatinine (Scr)-based formula of the full age spectrum (FAS) [7, 8]: 107.3/[Scr/Q] where

*Q* = 0.23 + 0.034 × age − 0.0018 × age^2^ + 0.00017 × age^3^ − 0.0000051 × age^4^, for girls < 18 years;

*Q* = 0.21 + 0.057 × age − 0.0075 × age^2^ + 0.00064 × age^3^ − 0.000016 × age^4^, for boys < 18 years; *Q* = 0.70 for female patients when age ≥ 18 years; *Q* = 0.90 for male patients when age ≥ 18 years

During recruitment, participants completed a validated food frequency questionnaire in which participants were asked to answer the question “How often, on average, in the past weeks did you eat [this food]?” These categorical answers were converted to average servings per week for analysis. Food intake frequencies were converted into conversion factors. Specifically, 1–3 servings/month was converted to 0.47, 1 serving/week converted to 1, and 2–4 servings/week converted to 3.

Data were expressed as median and interquartile range (IQR). Prevalence and 95% confidence interval (CI) of hyperuricemia were estimated overall and stratified by sex and age. Chi-square test was applied in group comparison. Binary logistic regression was used to assess the associations between hyperuricemia and the related risk factors in univariable and multivariable analyses. Student’s t test was applied in the comparison of food frequency. We did not impute missing data. Analyses were conducted using R statistics (version 3.4.16) and SPSS (version 19.0). *p* < 0.05 for a two-tailed test was considered statistically significant.

## Results

**Fig. 1:**
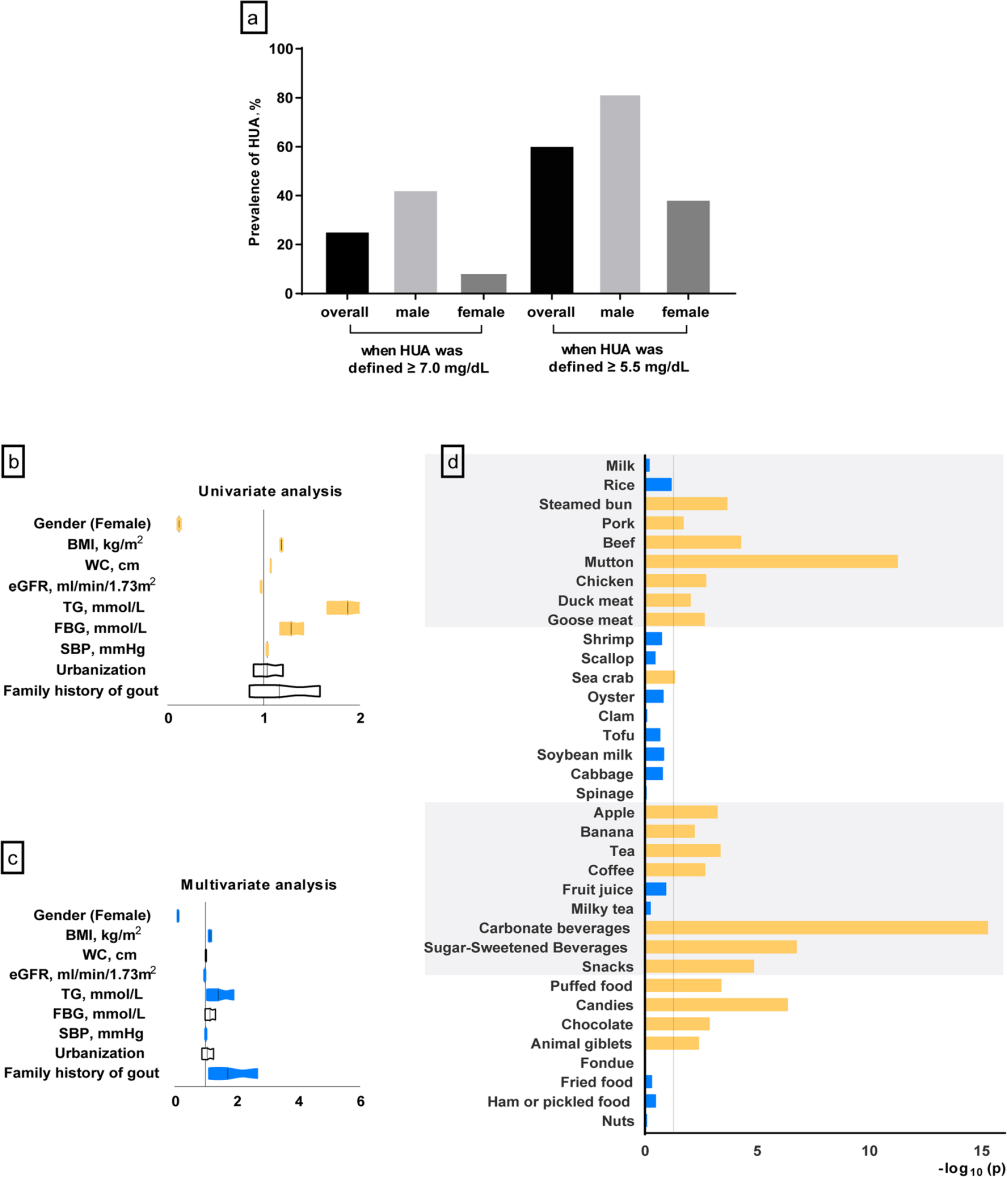
Summary of the hyperuricemia prevalence among Chinese adolescents and associated factors with hyperuricemia. sUA, serum uric acid; HUA, hyperuricemia; BMI, body mass index; WC, waist circumference; TC, total cholesterol; TG, triglycerides; eGFR, estimated glomerular filtration rate. **a** The prevalence of hyperuricemia among adolescents stratified by sex. Two standards of hyperuricemia were applied (sUA ≥ 7.0 mg/dL and sUA ≥ 5.5 mg/dL). ORs of risk factors associated with hyperuricemia (sUA ≥ 7.0 mg/dL) in univariate (**b**) and multivariate (**c**) analysis. BMI, WC, TG, FBG, SBP and eGFR are continuity variables. Gender (female/male), urbanization (urban/rural) and family history of gout (yes/no) are dichotomous variables. Multivariable logistic model was used to adjust for sex, BMI, WC, SBP, TG, FBG, eGFR, family history of gout and rural resident. Color bars show the significant differences (*p* < 0.05). **d** Manhattan plot of −log10 (*p*) for 35 food items associated with hyperuricemia. Bars in yellow represent the significant difference between hyperuricemia group and normal sUA group (*p* < 0.05). Bars in blue represent non-significance (*p* > 0.05)

The survey was completed by 9371 respondents out of the targeted 11,602 Chinese adolescents, giving the survey a good response rate of 80.8%. The final sample included 50.6% boys and 49.3% girls. The students’ ages ranged from 13 to 19 years old, with the median age of 16 years old. The general demographic and clinical characteristics were compared between hyperuricemia and normal sUA participants (Table 1). The overall median sUA level was 5.92 (IQR, 4.96–7.03) mg/dL (6.72 (IQR, 5.83–7.71) mg/dL for boys and 5.18 (4.47–5.97) mg/dL for girls). When hyperuricemia was defined as sUA ≥ 7.0 mg/dL, the overall hyperuricemia prevalence was 25.4% and prevalence in boys and girls were 42.3% and 8.0% respectively. When defined as sUA ≥ 5.5 mg/dL, the overall hyperuricemia prevalence was strikingly 60.5% and prevalence in boys and girls were 82.1% and 38.4% respectively (Fig. 1a).

**Table 1:** Demographic and general clinical characteristics of the participants according to sUA levels (hyperuricemia vs*.* normal sUA)

Variables	Total	HUA	Normal sUA	*P* value
N	9371	2282	7089	
Age, years	16 (15–18)	16 (15–18)	16 (15–18)	> 0.05
sUA, mg/dL	5.92 (4.96–7.03)	7.92 (7.41–8.71)	5.43 (4.69–6.17)	< 0.001
13	5.66 (4.74–6.77)	7.88 (7.38–8.45)	5.28 (4.55–6.03)	< 0.001
14	5.56 (4.74–6.69)	7.98 (7.42–8.65)	5.24 (4.54–5.97)	< 0.001
15	6.10 (5.14–7.18)	7.82 (7.41–8.64)	5.58 (4.82–6.25)	< 0.001
16	6.17 (5.28–7.36)	8.00 (7.51–8.89)	5.66 (4.98–6.25)	< 0.001
17	5.83 (4.84–6.92)	7.83 (7.41–8.57)	5.38 (4.59–6.20)	< 0.001
18	5.88 (4.96–7.07)	8.01 (7.36–8.69)	5.43 (4.66–6.22)	< 0.001
19	5.88 (4.96–6.97)	7.93 (7.37–8.80)	5.41 (4.67–6.12)	< 0.001
BMI, kg/m^2^	20.4 (18.5–23.0)	22.2 (19.6–22.1)	20.0 (18.3–26.0)	< 0.001
Urbanization				
Urban, *n* (%)	1866 (46.3)	431 (47.0)	1435 (46.1)	> 0.05
Waist circumference, cm	72.0 (67.0–79.0)	78.0 (71.0–88.0)	70.0 (66.0–77.0)	< 0.001
Obesity, *n* (%)	449 (4.8)	278 (12.2)	171 (2.4)	< 0.001
Hyperlipidemia, *n* (%)	317 (3.4)	117 (5.1)	200 (2.8)	< 0.001
Hyperglycemia, *n* (%)	47 (0.8)	17 (1.2)	30 (0.7)	> 0.05
Hypertension, *n* (%)	2037 (21.7)	733 (32.1)	1304 (18.4)	< 0.001
Metabolic syndrome, *n* (%)	20 (0.5)	17 (0.7)	3 (0.2)	< 0.001
eGFR, ml/min/1.73m^2^	102.2 (89.5–115.0)	95.3 (85.4–106.6)	104.7 (91.9–117.2)	< 0.001
≤75 ml/min/1.73m^2^, *n* (%)	490 (5.2)	142 (6.2)	348 (4.9)	0.014
Family history of gout, *n* (%)	212 (3.1)	56 (3.5)	156 (3.0)	> 0.05
*Menarche age, years	12 (12–13)	12 (11–13)	13 (12–13)	< 0.001
*Irregular menstruation, *n* (%)	1096 (41.2)	113 (45.2)	983 (40.8)	< 0.001

Data are presented as median (IQR, interquartile range). sUA, serum uric acid; HUA, hyperuricemia (sUA ≥ 7.0 mg/dL); BMI, body mass index; eGFR, estimated glomerular filtration rate. Obesity was defined as waist circumference ≥ 90th percentile among peer group; hyperlipidemia was defined as triglycerides ≥ 1.7 mg/dL; hyperglycemia defined as fasting blood glucose ≥ 5.6 mmol/L; hypertension ≥ 130 mmHg systolic or ≥ 80 mmHg diastolic; metabolic syndrome was defined as a proposed modification of the definition by the International Diabetes Federation. eGFR (ml/min per 1.73 m^2^) was calculated via the age-based formula of the Full Age Spectrum. Irregular menstruation delegates girls with irregular periods *Data were calculated based on females

Logistic regression analysis revealed male gender, obesity, and metabolic syndrome-related variables such as BMI, WC, TG, FBG, SBP were associated with the enhanced risk of hyperuricemia. Noticeably, positive family gout history was also found involved in the association of hyperuricemia risk by multivariate logistic regression analysis (Fig. 1b, c). Carbonate beverage, mutton, and sugar-sweetened beverage were the top three foods with a significant difference in consumption frequency between adolescents with hyperuricemia and with normal sUA (Fig. 1d). Food like steamed bun, pork, beef*,* etc. displayed significant differences in intake frequency as well (*p* < 0.05).

## Discussion

The current study sent an alarm of a pressing epidemic of hyperuricemia among adolescents in China. Considering reported prevalence (adults or overall population) of asymptomatic hyperuricemia is around 13.3% in China and between 4 and 25% in major countries of the world [3] and puberty uric acid (normally 3–4 mg/dL) levels are considerably lower than the adults [9], the results unequivocally revealed a hyperuricemia epidemic is happening in Shandong Province or beyond. We identified male gender, increased BMI, increased waist circumstance, increased TG, increased FBG, increased SBP, decreased eGFR, and positive family gout history were associated with the enhanced risk of hyperuricemia, which are similar with risk factors among adults [3].

In the 1999–2006 National Health and Nutrition Examination Survey, 6036 adolescents 12 to 17 years of age were included to analyze the relationship between uric acid and blood pressure [10]. In the adjusted analysis, the odds ratio of elevated blood pressure, for each 0.1 mg/dL increase in uric acid level was 1.38 (95% CI, 1.16 to 1.65). Compared with <5.5 mg/dL, participants with a uric acid level ≥ 5.5 mg/dL had a 2.03 fold (95% CI, 1.38 to 3.00) elevated blood pressure. Similarly, the proportion of hypertension in the hyperuricemia group was much higher than that in the normal sUA group and the elevated SBP was related to enhanced risk of hyperuricemia in our study. As we defined adolescent hypertension as ≥ 130 mmHg systolic or ≥ 80 mmHg diastolic by the 2017 AAP and 2017 ACC/AHA guidelines in this study, the overall prevalence of hypertension is high up to 21.7%. However, when we defined hypertension as ≥ 140 mmHg systolic or ≥ 90 mmHg diastolic using traditional recommendation, a less strict standard, the overall prevalence of hypertension is 7.3% in total (11.4% in hyperuricemia population and 5.9% in normal sUA ones).

High serum uric acid was positively associated with obesity in the overweight and obesity group in a cross-sectional study, which enrolled 3529 Chinese students aged 16–26 years old [11]. In another nested population cohort with a sample of 494 re-contacted adolescents from the original study, the prevalence of uric acid at risk was 37.25% and the proportion of high uric acid was 18.42%, significantly higher in men than in women. Adolescents with high levels of uric acid were more likely to have abdominal obesity (OR 3.03, 95% CI 1.38–6.64), hypertriglyceridemia (OR 4.94, 95% CI, 2.98–8.19), and altered fasting glycemia (OR 5.15, 95% CI, 3.42–11.05) [12]. Our data showed similar results that adolescents with enhanced BMI, increased TG, and increased FBG had higher probability of hyperuricemia occurrence in univariate and multivariate analysis.

The kidney is one of the main targets of hyperuricemia. In this study, we used the FAS-Age formula which is age based to indicate eGFR accordingly in adolescents. The current Kidney Disease Improving Global Outcomes (KDIGO) guidelines recommend the use of the bedside creatinine-based Chronic Kidney Disease in Children (CKiD) equation to estimate GFR in children and the Chronic Kidney Disease Epidemiology Collaboration (CKD-EPI) equation in adults. However, this approach causes implausible changes in eGFR at the transition from pediatric to adult care [8]. Compared with CKiD and CKD-EPI formula, FAS-Age was more accurate and more suitable for our adolescent participants. In our results, the decreased eGFR was also related to high hyperuricemia incidence in the logistic regression analysis.

Diet, especially purine-rich food, are risk factors for hyperuricemia. Recently, dietary fructose intake has been increasing. It is increasing primarily from added sugars, including sucrose and high-fructose corn syrup (HFCS), and correlates epidemiologically with the rising prevalence of hyperuricemia. A total of 814 adolescents aged 13–15 years participating in the Western Australian Pregnancy Cohort (Raine) Study showed fructose intake was independently associated with serum uric acid (*p* < 0.01) in boys [13]. The results from a pooled analysis indicated that total fructose consumption was positively associated with uric acid levels in adolescents [14]. Some cross-sectional analyses and clinical trials also supported the association between HFCS-sweetened beverage intake and increased levels of circulating uric acid [15–17]. Considering the mechanism, a study indicated that fructose-rich sugar-sweetened beverage (SSB) consumption is associated with an increase in pediatric insulin resistance through the pathways in relation to visceral fat accumulation and uric acid synthesis [18]. We showed there was a highly significant difference between carbonate and SSB in consumption frequency between adolescents with hyperuricemia and with normal sUA. This result was consistent with previously reported data. Further, controlling fructose-rich beverage intake would be an effective strategy in hyperuricemia prevention.

In addition, estrogen has been reported to inhibit hypoxia-induced xanthine oxidase activity and help the kidneys get rid of uric acid [19]. The significantly higher ratio of irregular menstruation in hyperuricemia girls than in normal sUA ones indicated lower levels of estrogen in hyperuricemia girls which need to be confirmed by further clinical test.

Several limitations of the study should be noted. First, the data are cross-sectional, and therefore it is not possible to establish that relatively higher uric acid levels preceded or caused increased BMI, increased waist circumstance, increased TG, increased FBG, increased SBP and decreased eGFR. Second, the representativity of the sample population is limited, though Shandong Province is one of the most populous provinces in China. Epidemic surveys of expanded population from other south provinces need to be conducted to delegate the Chinese hyperuricemia prevalence. Third, the limitation of estimating the GFR in adolescents and young adults. More accurate eGFR estimating equations should be induced to evaluate the renal functions.

This study expands the evidence that an increasing prevalence of hyperuricemia is shown in a cohort of 9371 Chinese adolescents in a cross-sectional study. Epidemic of youth hyperuricemia may pose a future threat of gout attacks and other hyperuricemia-related diseases, which alarms the public, health professionals and health policy makers to prepare for the future health challenges.
